# In Vitro Study of the Therapeutic Potential of Brown Crude Fucoidans in Osteoarthritis Treatment

**DOI:** 10.3390/ijms232214236

**Published:** 2022-11-17

**Authors:** Carlos Vaamonde-García, Emma Capelo-Mera, Noelia Flórez-Fernández, María Dolores Torres, Beatriz Rivas-Murias, Rosa Mejide-Faílde, Francisco J. Blanco, Herminia Domínguez

**Affiliations:** 1 Grupo de Investigación de Reumatología y Salud (GIR-S), Departamento de Biología, Facultad de Ciencias, CICA-Centro Interdisciplinar de Química y Biología, INIBIC-Sergas, Universidade da Coruña, Campus da Zapateira, 15011 A Coruña, Spain; 2 Grupo de Biomasa y Desarrollo Sostenible (EQ2), Departamento de Ingeniería Química, Facultad de Ciencias, CINBIO, Universidade de Vigo, 32004 Ourense, Spain; 3 CACTI, Universidade de Vigo, 36310 Vigo, Spain; 4 Grupo de Terapia Celular y Medicina Regenerativa, Universidade da Coruña, CICA-Centro Interdisciplinar de Química y Biología, Complexo Hospitalario Universitario A Coruña, Campus Oza, 15006 A Coruña, Spain; 5 Grupo de Investigación de Reumatología y Salud (GIR-S), Departamento de Fisioterapia, Medicina y Ciencias Biomédicas, Facultad de Fisioterapia, CICA-Centro Interdisciplinar de Química y Biología, INIBIC-Sergas, Universidade da Coruña, Campus de Oza, 15006 A Coruña, Spain

**Keywords:** osteoarthritis, proinflammatory cytokines, chondrocytes, crude fucoidans, nuclear factor-erythroid 2-related factor, hemeoxygenase 1, reactive oxygen species, senescence

## Abstract

Osteoarthritis, one of the most common joint degenerative pathologies, still has no cure, and current treatments, such as nonsteroidal anti-inflammatory drugs, can cause serious adverse effects when taken for a long time. Brown seaweed crude fucoidans are used for the clinical treatment of several pathologies. In this study, the therapeutical potential of these biocompounds was analyzed in primary chondrocytes and the 260TT human chondrocyte cell line. Crude fucoidan from *Undaria pinnatifida* (Up) and *Sargassum muticum* (Sm) was obtained by different extraction techniques (microwave-assisted extraction, pressurized hot-water extraction, ultrasound-assisted extraction) and chemically and structurally characterized by Fourier transform infrared spectroscopy, high-performance size-exclusion chromatography, proton nuclear magnetic resonance, and scanning electron microscopy. Once cell viability was confirmed in chondrocytes treated with crude fucoidans, we evaluated their anti-inflammatory effects, observing a significant reduction in IL-6 production stimulated by IL-1β. Findings were confirmed by analysis of IL-6 and IL-8 gene expression, although only fucoidans from Up achieved a statistically significant reduction. Besides this, the antioxidant capacity of crude fucoidans was observed through the upregulation of Nrf-2 levels and the expression of its transcriptional target genes HO-1 and SOD-2, with compounds from Up again showing a more consistent effect. However, no evidence was found that crude fucoidans modulate senescence, as they failed to reduced β-galactosidase activity, cell proliferation, or IL-6 production in chondrocytes stimulated with etoposide. Thus, the findings of this research seem to indicate that the tested crude fucoidans are capable of partially alleviating OA-associated inflammation and oxidative stress, but fail to attenuate chondrocyte senescence.

## 1. Introduction

Joints are an essential component of the musculoskeletal system. On the surface of the joints, there is articular cartilage, which allows the coupling and sliding of adjacent joint surfaces and bears the load during articular movement [1,2]. The hyaline cartilage consists of chondrocytes as unique cell type and an extracellular matrix (ECM) that these cells are responsible for maintaining [1,2,3]. Since hyaline cartilage is an aneural and avascular tissue, communication with cells and ECM occurs through the diffusion of molecules from synovial fluid, rendering the repair of the tissue difficult in case of damage [2,3]. The physical properties of this tissue depend mainly on its structure, which relies in turn on the integrity of its ECM and the chondrocyte morphology and function [1,4]; thus, any hyaline cartilage failure might lead to joint malfunction, the main feature of rheumatic diseases such as osteoarthritis (OA) [1].

OA is a joint degenerative pathology characterized by cartilage deterioration. Nevertheless, there are other tissues involved, such as the synovial membrane or the subchondral bone [5,6]. It is estimated that OA affects 250 million people worldwide, with the knee being the most common site of OA [6]. Moreover, women over 60 years of age have a higher probability of suffering from the disease. Likewise, the probability for an obese person of having knee, hip, or hand OA is four times greater in women and 4.8 times greater in men [7]. Therefore, several factors are involved in OA pathogenesis. Nonetheless, it is now accepted that an impairment in the function of the chondrocytes is a common and pivotal event in the development of this disease [1]. Different pathological events take place in the OA chondrocyte: senescence [8], mitochondrial dysfunction [9,10], apoptosis [1], over-activation of inflammasome [11], or impaired autophagy [12], among others. All of these may increase inflammation induced by cytokines, metalloproteinase (MMP) expression in the ECM, and reactive oxygen species (ROS) [8,10,11,12], thus leading to joint damage. Besides this, processes such as cellular senescence and mitochondrial dysfunction are also related to the imbalance in the antioxidant defense system and especially with the nuclear factor erythroid-2 related factor (Nrf-2)/heme oxygenase-1 (HO-1) axis [13,14].

The rise of proinflammatory cytokine expression in the cartilage, synovial membrane, and subchondral bone is linked to the development and progression of structural changes in the OA joint. The main proinflammatory cytokines associated with the pathophysiology of the disease are interleukin 1β (IL-1β), tumor necrosis factor α (TNF-α), and interleukin 6 (IL-6). These molecules are synthesized by chondrocytes, mononuclear cells, osteoblasts, and synovial tissues, inducing in turn the synthesis of other catabolic and inflammatory factors [15]. IL-1β is principally related to cartilage deterioration; it suppresses collagen type II and aggrecan synthesis; induces MMP 1, 3, and 13 release; and stimulates IL-6 and interleukin 8 (IL-8) production, which also activates pro-catabolic responses in the cartilage perpetuating joint degeneration and inflammation [15].

Cellular senescence is another important factor in OA pathogenesis [16,17]. Different studies show its connection with OA is related to age [18,19] or injuries [20,21], among others. Senescence is a stress response that causes a permanent arrest of the cell cycle and transcriptional, epigenetic, metabolic, and morphological changes leading to intense phenotypic changes. Senescent cells modify their environment in several ways, including by the senescence-associated secretory phenotype (SASP) [22,23]. SASP is the complex secretome of the senescent cells; some of its major components are IL-6, IL-17, IL-1β, and TNF [23].

At present, OA has no cure, and current treatments are based on the reduction in symptoms such as pain, inflammation, and cartilage degradation, and the preservation of joint mobility. Some of the drugs used to attenuate these symptoms, such as nonsteroidal anti-inflammatory drugs (NSAIDs) [6], cause serious adverse effects when are taken for a long time. Therefore, novel approaches must be found.

Brown algae crude fucoidans might be an alternative for the treatment of broader targets, since they contain therapeutic components, such as sulphated polymers, that show biological actions [24,25]. The sulphated polymer obtained from brown seaweeds can be extracted by several methodologies and technologies. Conventional methodologies, such as acid- or alkaline-based extractions, usually consume large volumes of solvents and chemicals and require prolonged extraction time, which could cause product degradation [26]. On the contrary, ecofriendly extraction technologies, such as ultrasound-assisted extraction, microwave-assisted extraction, or pressurized hot-water extraction, have several advantages, such as saving extraction time and resources, higher solubility, or higher yield when compared with conventional treatments [26]. Brown seaweeds, such as *Sargassum* spp. or *Undaria pinnatifida*, have garnered high interest from the scientific community. Their main polymer, fucoidan, has been associated with potential antioxidant, anti-inflammatory, antitumoral, antiviral, and antidiabetic biological properties and activities, among others [27,28,29].

Recent findings suggest that fucoidans may play an important role in OA treatment [30,31,32]. Nevertheless, since their composition is very diverse, no universal conclusions can be drawn, so that each type of fucoidan could work for a diverse therapeutic activity [33]. Fucoidans’ clinical effects have already been proven in several diseases such as cancer, neurological diseases, and diabetes [34], and some research suggests its application in rheumatic diseases [30,31]. Despite the fact that there is no approved treatment based on brown seaweed crude fucoidans for rheumatic diseases, some studies [32,35] and clinical trials [36] have already been carried out.

Based on the beneficial but diverse effects of brown seaweed crude fucoidans in the treatment of different pathologies and the previous findings of the group in rheumatic diseases [31,37], in this study we analyzed the therapeutical potential of crude fucoidans of *Undaria pinnatifida* (Harvey) Suringar (Up) and *Sargassum muticum* (Yendo) Fensholt (Sm) in an in vitro model of OA using cultured chondrocytes. The seaweeds and technologies have been selected based on previous studies for their high fucose and sulfate content and in vitro antioxidant activity [31,38,39,40]. Firstly, we performed a chemical and structural characterization of crude alga fucoidans obtained by different extraction techniques (Up-MAE: microwave-assisted extraction; Sm-PHW: pressurized hot-water extraction; Sm-US: ultrasound-assisted extraction), and subsequently evaluated and compared their anti-inflammatory, antioxidant, and anti-senescence effects in human primary chondrocytes and the chondrocyte cell line 260TT.

## 2. Results

### 2.1. Characterization of Crude Fucoidans 

Table 1 summarizes the fundamental composition of the tested crude fucoidans obtained from *Sargassum muticum* using both ultrasound-assisted extraction (Sm-US) [40] and pressurized hot-water extraction (Sm-PHW) [39] and from *Undaria pinnatifida* (Up-MAE) using microwave-assisted extraction [38]. The composition of the commercial fucoidan used for comparative purposes was also included [31]. Crude fucoidan from Sm-PHW and its commercial counterpart, Up-C, exhibited the highest polysaccharide content, with similar fucose and Gal+Xyl+Mn content.

#### 2.1.1. Fourier-Transform Infrared Spectroscopy (FTIR)

**Figure 1:**
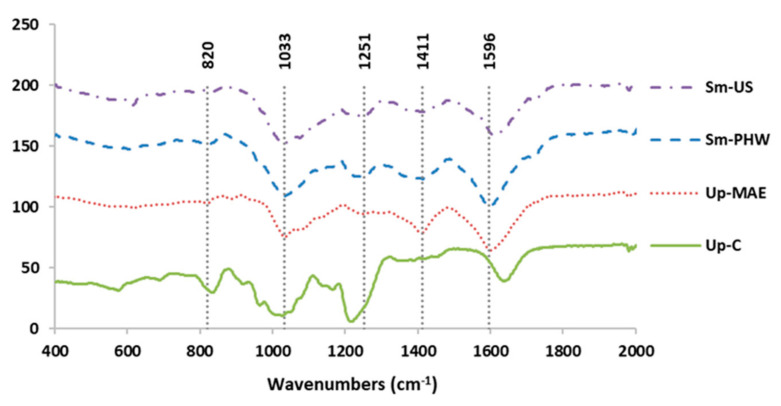
FTIR-ATR spectrum of crude fucoidan obtained from *Sargassum muticum* (Sm) from two extraction technologies: ultrasound-assisted extraction (Sm-US) and pressurized hot-water extraction (Sm-PHW), crude fucoidan from *Undaria pinnatifida* (Up-MAE), and commercial fucoidan from *Undaria pinnatifida* (Up-C) using microwave-assisted extraction [31,38,39,40].

As shown in Figure 1, FTIR spectra of the crude fucoidans ranging from 700 to 1700 cm^−1^ was performed to evaluate the main bands exhibited. Several common peaks at 820, 1033, 1251, 1425, and 1610 cm^−1^ were obtained despite two brown seaweeds being used, *S. muticum* and *U. pinnatifida*.

#### 2.1.2. High-Performance Size-Exclusion Chromatography

**Figure 2:**
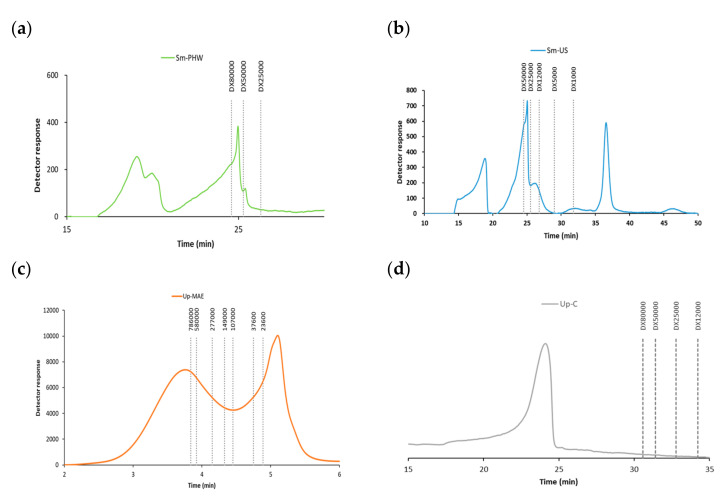
Detailed molar mass distribution profiles for crude fucoidan obtained from *Sargassum muticum* (Sm) using (**a**) pressurized hot-water extraction (Sm-PHW) and (**b**) ultrasound-assisted extraction (Sm-US), (**c**) the profile of the crude fucoidan obtained from *Undaria pinnatifida* (Up-MAE), and (**d**) the profile of the commercial fucoidan from *Undaria pinnatifida* using microwave-assisted extraction. Adapted from [31,38,39,40].

The molecular weight distribution of the tested crude fucoidans was represented in Figure 2. The crude fucoidans obtained from *S. muticum* by ultrasound-assisted extraction and pressurized hot-water extraction were represented in Figure 2a,b, respectively.

The profile of Sm-US exhibited three peaks: under 1000 Da, between 50 kDa to 12 kDa, and a higher peak above 50,000 Da. On the other hand, the crude fucoidan obtained by pressurized hot-water extraction (Sm-PHW) showed two peaks, between 80–50 kDa and one above 80 kDa. The crude fucoidan of *U. pinnatifida* obtained by MAE is displayed in Figure 2c, exhibiting two peaks: under 23.6 kDa and above 58 kDa. In addition, the commercial fucoidan of *U. pinnatifida* is shown in Figure 2d.

#### 2.1.3. Proton Nuclear Magnetic Resonance (^1^H NMR)

**Figure 3:**
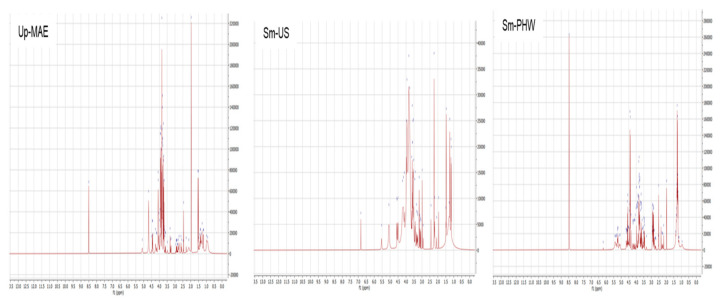
^1^H NMR spectra of crude fucoidan from *Sargassum muticum* (Sm) after ultrasound-assisted extraction (Sm-US) and pressurized hot-water extraction (Sm-PHW), and crude fucoidan from *Undaria pinnatifida* (Up-MAE) after microwave-assisted extraction.

The proton nuclear magnetic resonance spectra for the three crude fucoidans studied are shown in Figure 3. The ^1^H NMR spectra comparison indicated some differences between the crude fucoidans. A higher signal intensity was identified for crude fucoidans from both hydrothermal treatments (Sm-PHW and Up-MAE crude fucoidans), with a smaller signal magnitude for Sm-US crude fucoidan (about fivefold). 

A band at 8.5 ppm was observed for Sm-PHW and Up-MAE crude fucoidans, which seems to appear more weakly at 7.0 ppm for Sm-US. In all cases, abundant and diversified peaks are found between 3.0–5.5 ppm and 2.0–2.5 ppm and some between 1.0–1.5 ppm.

#### 2.1.4. Scanning Electron Microscope (SEM)

**Figure 4:**
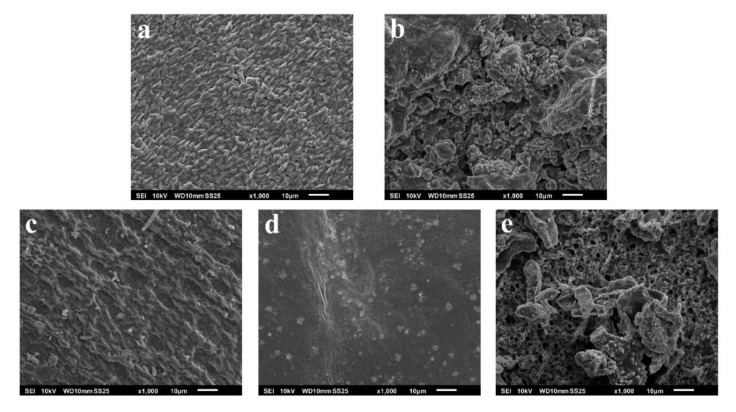
SEM images of Up (**a**), Up-MAE (**b**), Sm (**c**), Sm-PHW (**d**), and Sm-US (**e**) samples.

The surface morphology of the seaweed samples was studied by SEM (Figure 4). The morphology of the untreated seaweed (1a and 1c) shows a regular mosaic-like pattern for the Up and Sm samples. After the different treatments, the morphology of the seaweed changes notably. In the case of the Up-MAE crude fucoidan (1b), the initial pattern breaks up and a more irregular one appears with larger roughness, together with the presence of small crystalline precipitates containing sodium from the extraction process. For the Sm-PHW and Sm-US crude fucoidans, the initial regular morphology disappears, being more pronounced for the pressurized hot-water extraction sample, where the surface became smoother. This feature clearly points to the fact that this method is more aggressive than the others.

### 2.2. Effect of Crude Fucoidans on Cell Viability and Inflammatory Response in Human Chondrocytes

**Figure 5:**
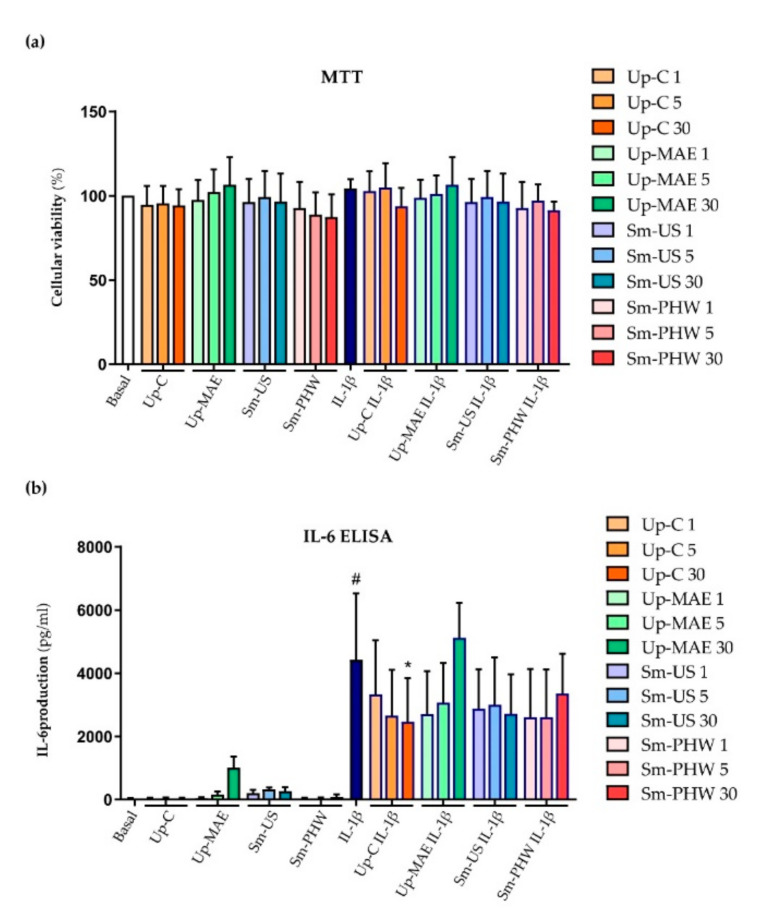
Effect of crude fucoidans and *U. pinnatifida* commercial fucoidan on cell viability (**a**) and inflammatory response (**b**) induced by IL-1β in human primary chondrocytes. (**a**) Cell viability was measured by MTT assay (*n* = 4) within the different experimental conditions: non-treated (basal) and treated with IL-1β or Up-C, Up-MAE, Sm-US, and Sm-PHW at three different concentrations (1, 5, and 30 µg/mL) with and without IL-1β. (**b**) IL-6 production was measured at protein level by IL-6 ELISA (*n* = 4) (**a**) in cells stimulated as previously indicated for 24 h. IL-1β condition is statistically different from basal condition (#: *p*-value < 0.05). Conditions marked with * are statistically different from IL-1β condition (*: *p*-value < 0.05).

The toxic effects of the three algae extracts were tested in human primary OA chondrocytes in the absence and presence of IL-1β, a known inductor of inflammatory response in chondrocytes. Besides this, cells were also treated with fucoidan from *Undaria pinnatifida* (Up-C) as a safe control based on previous studies [31,37]. After performing an MTT cell-viability assay (*n* = 4), we observed that none of the brown algae extracts nor the tested commercial fucoidan affected cell viability (Figure 5a).

Once the safety of the treatment with both the algae extracts and the commercial fucoidan was demonstrated in human primary chondrocytes, IL-6 ELISA was performed to test its modulatory effects on the inflammatory response (Figure 5b). As expected, treatment with IL-1β significantly increased the IL-6 production. Interestingly, lower concentrations of crude fucoidans and all doses of the tested commercial fucoidan appeared to attenuate IL-1β-induced IL-6 levels, achieving significant modulations only for Up-C at 30 µg/mL (Figure 5b).

### 2.3. Effect of Crude Fucoidans on Cell Viability and Inflammatory Response in Chondrocyte Cell Line

**Figure 6:**
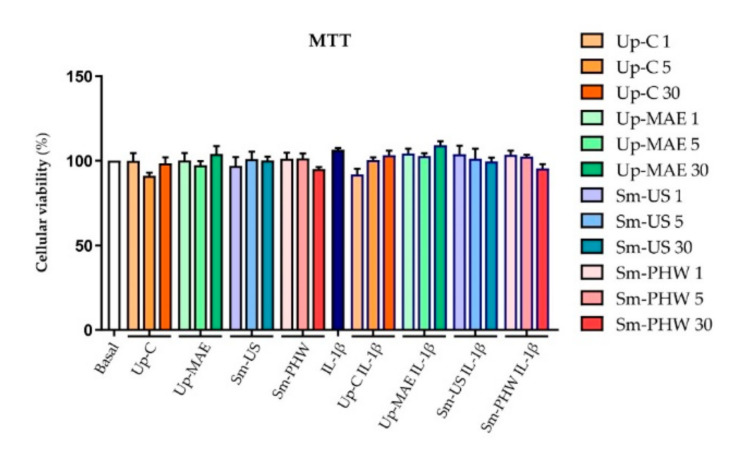
Effect of crude fucoidans and *U. pinnatifida* commercial fucoidan on cell viability of 260TT cell line. Cell viability was measured by MTT assay (*n* = 4) within the different experimental conditions: non-treated (basal), and treated with Up-C, Up-MAE, Sm-US, and Sm-PHW at three different concentrations (1, 5, and 30 µg/mL) with and without IL-1β. There are no significant differences between any of the conditions respecting the basal one nor the one stimulated only with IL-1β (*p*-value > 0.05).

To further investigate the above results and due to our limitation in obtaining primary human OA chondrocytes, we performed the following experiments in human immortalized 260TT chondrocytes, previously obtained in our lab [41]. First, the toxicity of the three crude fucoidans and commercial fucoidan as control was tested in the 260TT cell line. By MTT cell-viability assay (*n* = 4), we again found that there is no statistical evidence that the crude fucoidans nor the commercial fucoidan affect cell viability (Figure 6).

**Figure 7:**
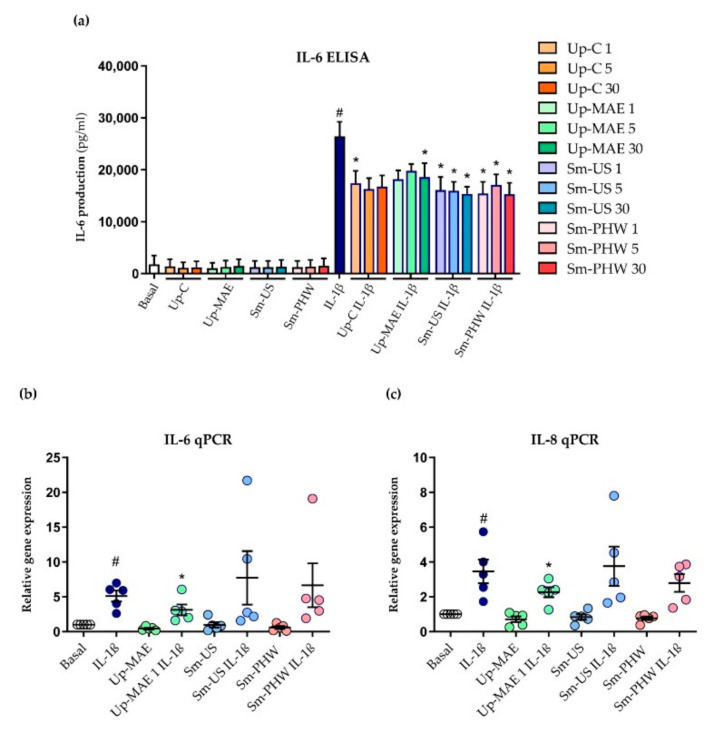
Effect of crude fucoidans on inflammatory response induced by IL-1β in 260TT cell line. (**a**) IL-6 production was measured at protein level by IL-6 ELISA (*n* = 4) in cells stimulated as previously indicated for 24 h. IL-6 (**b**) and IL-8 (**c**) gene expression was analyzed by qPCR (*n* = 4) in cells treated with crude fucoidans at 5 µg/mL with and without IL-1β for 6 h. IL-1β condition is statistically different from basal condition (#: *p*-value < 0.05). Conditions marked with * are statistically different from IL-1β condition (*: *p*-value < 0.05).

Once the safety of the treatment with both the crude fucoidans and the commercial fucoidan was demonstrated, IL-6 ELISA was performed to test their effect on the inflammatory response in 260TT cells. As we can see in Figure 7a, all the substances at every concentration reduced the IL-6 production induced by positive control, IL-1β. However, only the reductions by Sm-US and Sm-PHW are statistically significant at every dose; Up-C and Up-MAE are significant at 1 and 30 µg/mL, respectively. We then proceeded with qPCR analysis to confirm the results at the gene level. We analyzed IL-6 and IL-8 expression elicited by IL-1β in the presence of the intermediate concentration of each crude fucoidan, 5 µg/mL. As observed in Figure 7b,c, only Up-MAE significantly downregulated the expression of both inflammatory mediators.

### 2.4. Effect of Crude Fucoidans on Antioxidant Response

**Figure 8:**
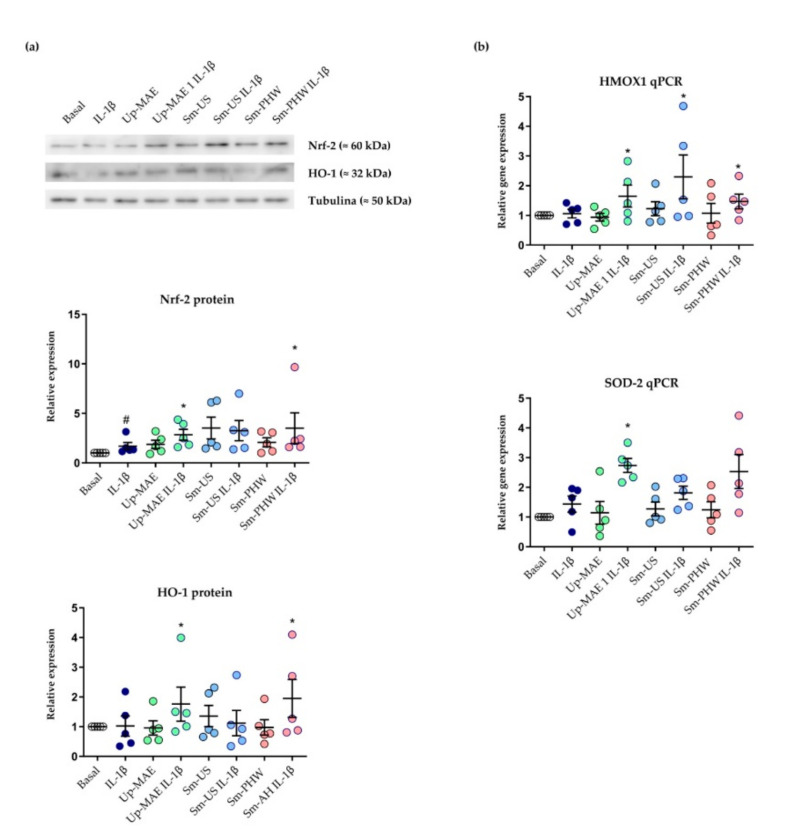
Effect of crude fucoidans on antioxidant response of 260TT cell line. (**a**) Nrf-2 and HO-1 protein levels were measured by Western blot (*n* = 4) in cells treated with crude fucoidans at 5 µg/mL with and without IL-1β for 24 h. Protein band intensities were quantified by densitometry with ImageQ image-processing software and relative expression of targeted protein was calculated and normalized by values of its related tubulin band intensity. (**b**) gene levels of HMOX-1 and SOD-2 were assessed by qPCR (*n* = 4) in cells treated with crude fucoidans at 5 µg/mL with and without IL-1β for 6 h. IL-1β condition is statistically different from basal condition (#: *p*-value < 0.05). Conditions marked with * are statistically different from IL-1β condition (*: *p*-value < 0.05).

The next step was testing whether the anti-inflammatory effect of the crude fucoidans could be due to their capacity to activate the antioxidant response. For this purpose, we evaluated the protein expression of the Nrf-2 and HO-1 components of the Nrf-2/HO-1 antioxidant pathway. We observed that Up-MAE and Sm-PHW significantly upregulated the protein levels of both Nrf-2 and HO-1 (Figure 8a).

To confirm our results, we analyzed the expression of HMOX1 and SOD-2 genes as indicators of Nrf-2 transcriptional activity with qPCR analysis. Cells treated with Up-MAE significantly upregulated SOD-2 gene levels induced by IL-1β, whereas in the case of HMOX1, all tested crude fucoidans increased its gene expression under IL-1β stimulation (Figure 8).

### 2.5. Effect of Crude Fucoidans on Cellular Senescence Modulation

**Figure 9:**
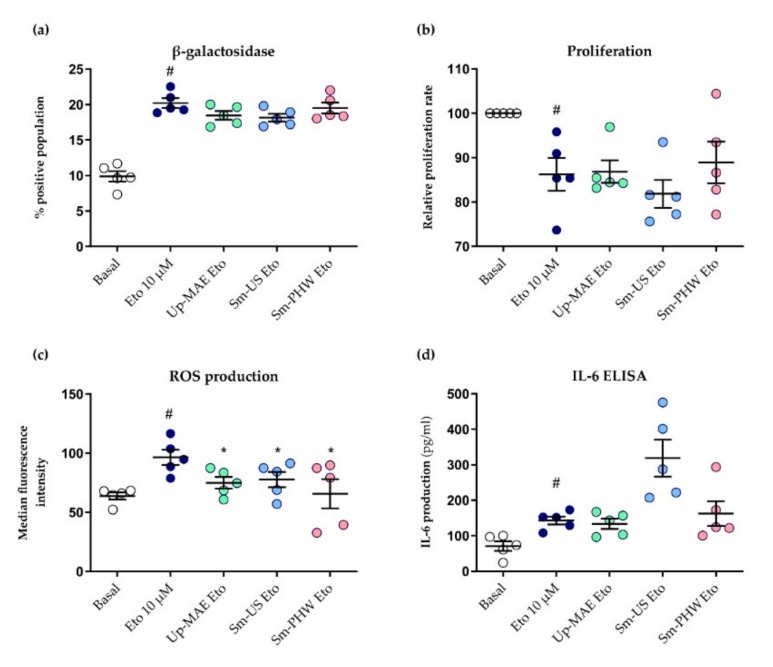
Effect of crude fucoidans on cellular senescence induced by etoposide in 260TT chondrocytes. Cells were treated as indicated for 48 h. Following this, (**a**) abundance of senescent chondrocytes was assessed through measurement of β-galactosidase activity by flow cytometry (*n* = 5), (**b**) proliferation ratio was evaluated by BrdU proliferation assay (*n* = 5), (**c**) ROS production was monitored by flow cytometry (*n* = 5), and (**d**) IL-6 production was measured by IL-6 ELISA (*n* = 5). Etoposide condition is statistically different from basal condition (#: *p*-value < 0.05). Conditions marked with * are statistically different from etoposide condition (*: *p*-value < 0.05).

Finally, we also evaluated whether the crude fucoidans could modulate activation of different pathways involved in the cellular senescence of chondrocytes, a process known to be involved in OA pathogenesis [16,17]. For this purpose, the 260TT chondrocyte cell line was stimulated with etoposide 10 µM, a well-known senescence inducer [42]. First, flow cytometry was performed to assess β-galactosidase activity through monitoring the fluorescent signal resulting from fluorescein di-β-galactopyranoside (FDG) dissociation triggered by the enzyme. As expected, etoposide increased the fluorescent signal, i.e., β-galactosidase activity, significantly, an indicator of senescent cells [43]. However, none of the crude fucoidans decreased enzyme activity in a consistent and statistically significant manner (Figure 9a). 

Cellular senescence is also characterized by a loss of proliferation capacity. Thus, a BrdU proliferation assay was developed to test if the crude fucoidans were able to restore the ability of the cells to grow at a normal rate. Etoposide significantly reduced the proliferation rate, but no evidence was found that the crude fucoidans restored the attenuated proliferation rate (Figure 9b). 

ROS production, an important component of the SASP, was assayed in the chondrocytes by flow cytometry. As shown in Figure 9c, all the crude fucoidans significantly decreased ROS production induced by etoposide. Besides this, IL-6 ELISA was carried out to test whether the crude fucoidans were able to reduce the synthesis of IL-6 as another component of the SASP. Contrary to what was observed in the case of IL-6 as part of the inflammatory response, we found no evidence that the crude fucoidans could decrease the production of IL-6 induced by etoposide (Figure 9d).

## 3. Discussion

In this study, we explore a novel strategy to face OA symptoms, testing the therapeutic effect of crude fucoidans in OA physiopathology, and observing an anti-inflammatory effect and the capacity to activate antioxidant responses. However, the tested crude fucoidans appear to be ineffective against senescence in chondrocytes.

First, we characterized the crude fucoidans chemically and structurally in order to determine differences between them. The results related to FTIR spectra (Figure 1) were consistent with previous works where different varieties of *Sargassum* were used to extract crude fucoidans employing mild acid-extraction conditions. In both studies, the bands associated with carbohydrates such as fucose, mannose, glucose, and xylose were observed [44]. According to the literature, the band exhibited at 820 cm^−1^ corresponds to C-O-S bending vibrations, while the band acquired at 1033 cm^−1^ is associated with the symmetric vibration C-O, related to C-O-SO_3_ group [45]. Besides this, the peak obtained at 1255 cm^−1^ is related to S=O stretching [46]. In the case of the band obtained at 1425 cm^−1^, this wavenumber has been designated to asymmetrical bending vibrations from fucose, glucose, mannose, and xylose [44]. The peak shown at 1610 cm^−1^ represents the asymmetric stretching vibrations associated with the carboxylate O─C─O bond [46].

The effect of the severity of the three employed technologies was found in the molar mass distribution of the tested crude fucoidans (Figure 2). In the case of the seaweed *S. muticum*, the obtained peaks showed fractions with different molecular masses depending on the extraction technology applied. *U. pinnatifida* algae presented a high molecular mass profile. Several authors have studied the relation between the activity and molecular weight of sulphated polysaccharides obtained from seaweeds, observing a relation between the activity and the molecular weight [47,48,49]. Accordingly, the antioxidant actions of the crude samples were more consistent in *U. pinnatifida* crude fucoidan (Figure 8).

Structural analysis (Figure 3) highlighted macromolecule variations depending not only on the studied brown seaweed species but also on the selected extraction technology. Differences on chemical and structural footprinting were previously reported for *S. muticum* collected from different geographical locations [50] and *Undaria pinnatifida* [51]. Latter authors reported phenolic compounds at around 6.0–9.0 ppm, suggesting that in the present study Sm-PHW featured the highest values of this fraction, followed by Up-MAE and Sm-US. The peaks between 3.0–5.5 ppm, which were related to polyols described as mannitol in tested algae [52], were more prominent for Up-MAE. The ratio of the amino acid glutamine/glutamates versus the malic acid/malates (towards 2.0–2.5 ppm) is pointed out for Up-MAE [51]. In contrast, some unsaturated fatty acids (1.0–1.5 ppm) were more abundant in Sm-PHW [50]. Interestingly, previous studies have observed that fatty-acid-rich lipid extracts from brown algae, such as *Sargassum*, present anti-inflammatory and antioxidant activity [53,54], which could explain the results detected in the current study.

One of the main concerns about OA treatments is their safety for human health. In the present study, it has been proven that the crude fucoidans do not affect cell viability (Figure 5 and Figure 6). Similarly, different studies testing crude fucoidans have observed safe and protective effects against toxic compounds at the same concentration tested in our study [55,56]. Additionally, these results are similar to those found by Vaamonde García et al. in 2021 [37] using brown algae commercial fucoidans at similar concentrations in human chondrocytes.

Inflammation is considered one of the most outstanding factors involved in OA pathogenesis [15]. In previous research from Jeon et al. in 2019, it was demonstrated in vivo as well as in vitro that extracts from *Sargassum muticum* decrease IL-6 and TNF-α synthesis in mice suffering from rheumatoid arthritis (RA). Besides this, they showed evidence of lower articular surface damage in mice treated with the extract than in RA mice by histopathological analysis [35]. Similarly, anti-arthritic actions of fucoidans from *U. pinnatifida* have also been observed [57]. In the current study, we evaluated the anti-inflammatory effect of the extracts on IL-6 and IL-8 (Figure 5 and Figure 7). At the protein level, it has been proven that both crude fucoidans from *U. pinnatifida* and *S. muticum* reduce IL-6 production. However, we detected that *U. pinnatifida* crude fucoidans, but not those from *S. muticum*, decreased both IL-6 and IL-8 expression at gene levels. Thus, *U. pinnatifida* was the crude fucoidan that showed the most consistent and significant effect against inflammation (Figure 7). Likewise, in previous research of the group [37], the anti-inflammatory effect of *U. pinnatifida* commercial fucoidan was proven—the one used in this study as a control.

The Nrf-2/HO-1 signaling system is a pivotal pathway activated in the cell to counteract oxidative stress and inflammation [58,59]. Nonetheless, different findings suggest that this antioxidant system is compromised in OA chondrocytes [60,61]. Likewise, different studies carried out in OA and arthritic chondrocytes have observed that *U. pinnatifida* fucoidan and *Sargasum* extracts show antioxidant actions and attenuating activation of pathological pathways in the articular cells [25,57]. Likewise, evidence in other types of cells and tissues suggest that fucoidans from brown algae including *Sargassum* or *Fucus* suppress oxidative stress by regulation Nrf-2/HO-1 signaling [62,63]. Thus, in the current study, we evaluated the capacity of the crude fucoidans to active antioxidant responses through the upregulation of Nrf-2/HO-1 expression in chondrocytes (Figure 8). Crude fucoidans from *U. pinnatifida* and only *S. muticum* obtained by subcritical water extraction significantly increased protein levels of Nrf-2 and HO-1, an antioxidant enzyme classically considered a reliable hallmark of Nrf-2 target gene. Accordingly, the same extracts upregulated the gene expression of HO-1. However, only *U. pinnatifida* significantly increased SOD-2 gene levels. In this regard, there is some evidence for the Nrf-2-independent regulation of SOD-2 [64] that could explain the result obtained in our study. Other proteins that play an important role against ROS are FoxOs transcription factors, which are involved both in the upregulation of antioxidant enzymes, such as SOD-2, and the modulation of key factors involved in cartilage homeostasis, such as vascular endothelial growth factor expression [65,66]. Thus, these proteins will deserve attention in future studies.

Another important factor involved in OA is cellular senescence [67]. Β-galactosidase is a well-known senescence marker [68] and its expression in articular cartilage is associated with joint damage typical of OA; thus, it is a sign of the degree of the disease [43]. Besides this, an additional feature of cellular senescence is cell-cycle arrest. In this study, we induced senescence in chondrocytes by stimulation with etoposide, observing a higher β-galactosidase activity and reduced proliferation ratio (Figure 9). The treatment with the crude fucoidans failed to attenuate these processes. Conversely, recent research [69] demonstrated an anti-senescence effect of extracts from brown algae *Ecklonia stolonifera* in human dermal fibroblasts. However, these authors used nanoparticles to pierce the extract into the cell, which might be a more efficient mechanism so the effect might be greater. Future research is warranted to elucidate this issue.

Senescent cells are also characterized by increased ROS production, mostly produced by dysfunctional mitochondria [70]. The stimulation of cells with etoposide increased ROS production, but treatment with all crude fucoidans could decrease it significantly (Figure 9). Similarly, previous studies testing commercial fucoidans in chondrocytes also reduced ROS production in cells treated with an inhibitor of mitochondrial electron transport and IL-1β [25,37]. Finally, the modulation of IL-6 synthesis as a component SASP phenotype, that is, the analysis of IL-6 production stimulated by etoposide instead of IL-1β in chondrocytes, was also analyzed. In these experiments, etoposide induced a significant but small increment in IL-6 production. However, no evidence was found that the crude fucoidans could attenuate it, and Sm-US seemed even to increase IL-6 levels (Figure 9). This could be due to the different modulation of IL-6 production, whether this is induced by IL-1β or etoposide; thus, further studies will be needed. 

In summary, our results indicate the biological activity of crude fucoidans from brown algae could vary depending on origin and extraction method. Likewise, we observed that crude fucoidan can modulate inflammatory and oxidative responses by attenuating proinflammatory cytokine production and activating the antioxidant system Nrf-2/HO-1. In addition, we have also demonstrated the capacity to directly attenuate ROS production. However, we found no evidence crude fucoidans can modulate cellular senescence. Future research is required to confirm the results obtained and to further elucidate issues raised in the current study.

## 4. Materials and Methods

### 4.1. Raw Materials for Crude Fucoidan Extraction

*Sargassum muticum* brown seaweed was collected in 2016 (August) in Pontevedra (42.24176° N, 8.771932° W, Spain). The alga was washed at the laboratory with tap water, ground, and stored in sealed plastic bags in darkness at −18 °C until use. *Undaria pinnatifida* brown seaweed was purchased from Algamar (Pontevedra, Spain). The raw material was stored in sealed plastic bags, in darkness and at room temperature. The fundamental compositional analysis for both algae has been previously reported [38,39]. Commercial fucoidan was purchased from Sigma-Aldrich (San Luis, CA, USA).

### 4.2. Extraction Technologies

#### 4.2.1. Ultrasound-Assisted Extraction (US) 

*S. muticum* was mixed with distilled water at a 1:20 (*w*/*w*) solid:liquid ratio. The extraction was conducted in an ultrasonic bath (P-Selecta, Spain) at room temperature. The operating conditions were 1.5 A intensity, 150W power, and 40 Hz frequency for 25 min. According to Flórez Fernández et al. in 2017 [40], two phases (liquid and solid) were separated after centrifugation. 

#### 4.2.2. Pressurized Hot-Water Extraction (PHW)

Hydrothermal treatment of *S. muticum* brown alga was performed mixing the alga with distilled water at a solid:liquid ratio of 1:30 (*w*/*w*) and introduced in a stainless steel reactor (Parr Instr., IL, USA). The suspension was non-isothermally heated up to 170 °C; therefore, when the temperature was achieved, the stainless steel reactor cooled down quickly and the suspension was separated by filtration. The alginate of the liquid phase was precipitated using CaCl_2_ at 1% (*w*/*w*, Acros Organics, Geel, Belgium), and split by centrifugation (4500 rpm, 40 min, Rotixa 50RS, Hettich Zentrigugen, Germany). 

#### 4.2.3. Microwave-Assisted Extraction (MAE)

The ground seaweed *U. pinnatifida* was introduced in a microwave-assisted device (Anton Paar Microwave reactor Monowave 450, Graz, Austria), using a solid/liquid ratio 1:30 (*w*/*w*), at 160 °C, rotation speed of 800 rpm. After treatment, the samples were cooled down until 50 °C [71]. Liquid and solid phases were separated by filtration. The alginate of the corresponding liquid fraction was precipitated using CaCl_2_ (Sigma-Aldrich, San Luis, CA, USA) at 1% (*w*/*w*) with stirring overnight at room temperature, producing the alginate-free liquid phase used for further analysis.

### 4.3. Fourier-Transform Infrared Spectroscopy (FTIR)

The crude fucoidans obtained by both US and PHW from *S. muticum* and the extract recovered after MAE from *U. pinnatifida* were lyophilized. Subsequently, the crude fucoidans and the commercial fucoidan were blended with potassium bromide and dried with an infrared lamp (30 min). The spectra were recorded at 400–4000 nm (25 scans/min), the device used being a Bruker IFS 28 Equinox (OPUS-2.52 software for data acquisition System 450-MT2). 

### 4.4. High-Performance Size-Exclusion Chromatography (HPSEC)

The molar mass distribution was performed to assess the molecular weight distribution of the crude fucoidans obtained from the two algae by the three ecofriendly intensified extraction technologies. The molar mass distribution profile was also evaluated for *U. pinnatifida* commercial fucoidan. The HPLC consisted of two columns in series TSKGel G3000PWXL and TSKGel G2500PWXL (300 × 7.8 mm, Tosoh Bioscience, Stuttgart, Germany), with a PWX guard column (40 × 6 mm). The operation conditions were Milli-Q water at 0.4 mL/min as a mobile phase at 70 °C, the standard being dextran from 1000–80,000 Da (Fluka, Newport News, VA, USA). This system was used to study the crude fucoidans obtained from *S. muticum*. On the other hand, the crude fucoidan obtained from *U. pinnatifida* was determined using a SuperMultipore PW-H column (6 mm × 15 cm) with a guard column SuperMP (PW)-H (4.6 mm × 3.5 cm, TSKgel Tosoh Corporation, Tokio, Japan). The selected operation conditions to work with this equipment were 40 °C, Milli-Q water as a mobile phase at 0.4 mL/min, and a refractive index detector. Polyethylene oxide at different molecular weights (23,600–786,000 Da) was used as a standard (Tosoh Corporation, Japan).

### 4.5. Proton Nuclear Magnetic Resonance (^1^H NMR)

The crude fucoidans of the two seaweeds obtained by three different extraction technologies were analyzed by proton nuclear magnetic resonance (1H NMR), using a Bruker ARX400 spectrometer (Bruker BioSpin GmbH, Billerica, MA, USA). The employed internal standard was 3-(trimethylsilyl)-L-propane sulfonic acid (Sigma-Aldrich, San Luis, CA, USA) and the solvent was deuterated water. The operation conditions were 400 MHz and 75 °C.

### 4.6. Scanning Electron Microscope Analysis (SEM)

A scanning electron microscope (Jeol JSM6010LA, Japan) was used to characterize the surface morphology of the raw samples and the residual solids after the different treatments. The samples were fixed on an adhesive carbon tape and coated with gold (15 nm). Secondary electron (SE) images were taken at an accelerating voltage of 10 kV and at several magnifications.

### 4.7. Cell Culture and Stimulation of Human Articular Chondrocyte 

To perform the study, both OA human primary chondrocytes and immortalized human chondrocyte cell line were used. Human chondrocytes were obtained as previously described from the hip joints of 4 adult female donors (mean ± SD age 78 ± 9 years) [61]. Likewise, informed consent was obtained for experimentation with human samples. Subcultures of isolated cells were performed with trypsin (Gibco Life Technologies, UK), after second-passage chondrocytes were used for experiments. Immortalized human chondrocyte cell line 260TT was obtained in our lab by Piñeiro Ramil et al. [41] and employed at different passages.

Cells were cultured in monolayer in Dulbecco’s modified eagle medium (DMEM) (Lonza, Basel, Switzerland) with 10% fetal bovine serum (FBS) (Gibco, Life technologies, Pasley, UK) and 1% penicillin/streptomycin (P/S) and were maintained in an incubator at 37 °C, 5% CO_2_ and with a moist atmosphere (95% humidity). Once cells reached confluence, cells were subcultured and used for the different experiments. To test the effect of the crude fucoidans on the inflammatory response and on the cellular senescence modulation, we established an inflammatory and a senescence model, which consisted of stimulating cells with either IL-1β (Sigma-Aldrich, San Luis, CA, USA) at 5 ng/mL or etoposide (Sigma-Aldrich, San Luis, CA, USA) at 10 µM, respectively, as positive controls. Once subcultures reached confluence, cells were made quiescent by 48-h incubation in either 0.5% DMEM (inflammatory model) or 2% DMEM (senescence model). Afterwards, cells were stimulated with the crude fucoidans at 0% DMEM (inflammatory model) or 2% DMEM (senescence model) at three different concentrations: 1, 5, and 30 µg/mL. All studies were performed strictly in accordance with Galician Research Ethics Committee regulations and the Declaration of Helsinki. 

### 4.8. MTT Viability Assay

Cells were incubated in a 96-well plate (10,000 cells/well) and stimulated with IL-1β and the crude fucoidans for 24 h. Cellular viability was then evaluated by the capacity of the chondrocytes to reduce the 3-(4,5-dimethylthiazol-2-yl)-2,5-diphenyltetrazolium bromide (MTT) to formazan using MTT Cell Assay Kit (Sigma-Aldrich, San Luis, CA, USA). Formazan crystals were then dissolved by adding sodium dodecylsulfate (SDS) and incubated overnight. The following day the absorbance was measured at 570 nm in a spectrophotometer (NanoQuant). The relative cell viability was represented by the percentage of absorbance in each experimental condition in relation to those values obtained in basal condition (100%).

### 4.9. ELISA

Chondrocytes were incubated in a 96-well plate (10,000 cells/well) and stimulated as indicated for 24 h. To analyze the content of IL-6 among the different conditions, an enzyme-linked immunosorbent assay (ELISA) commercial kit for IL-6 detection (Bio-Techne R&D Systems, Madrid, Spain) was performed. To analyze the results, the absorbance was measured at 450 nm in a spectrophotometer (NanoQuant).

### 4.10. RNA Analysis

Cells were incubated in a 12-well plate (70,000 cells/well) and stimulated as indicated for 24 h. First, RNA was extracted from the chondrocytes with TRIzol (Invitrogen, Thermo Fisher Scientific, Waltham, MA, USA) and chloroform (Sigma-Aldrich, San Luis, CA, USA). RNA was quantified in a NanoDrop^TM^ spectrophotometer (Thermo Scientific, Madrid, Spain) at 260 nm. Following this, mRNA was retrotranscribed using 500 ng of extracted RNA by a NZY First-Strand cDNA Synthesis kit (Nzytech, Lisbon, Portugal) in a thermocycler (Applied Biosystems, Thermo Fisher Scientific) [31]. Complementary deoxyribonucleic acid (cDNA) was amplified and quantified by quantitative polymerase chain reaction (qPCR) for 45 cycles in a LightCycler (Roche diagnostics, Basilea, Switzerland) using SYBR Green I Master (Roche Diagnostics, Sigma-Aldrich, San Luis, CA, USA) and gene-specific primers shown in Table 2. The relative gene expression was calculated and normalized to the relative levels of the hypoxanthine phosphoribosyl transferase (HPRT) mRNA using the 2(-Delta Delta C(T)) method.

### 4.11. Western Blot

Chondrocytes were incubated in a 12-well plate (70,000 cells/well) and stimulated with IL-1β and the crude fucoidans for 24 h. Thereafter, the cells were lysed by adding 50 µL of lysis buffer (Tris-HCl 0,2M pH 6,8; SDS 2%; glycerol 20%; phenylmethylsulphonyl fluoride (PMSF) 1% (Sigma-Aldrich, San Luis, CA, USA); inhibitors cocktail 1%) in each well and scrapped [61]. Protein extracts were then quantified in a NanoDrop^TM^ spectrophotometer at 280 nm. Subsequently, 20 µg of protein extracts were separated by electrophoresis in a 10% acrylamide/ bisacrylamide gel under reducing (β-mercaptoethanol) and denaturing (SDS) conditions at 80V, so that proteins migrated only according to their molecular weight. Once electrophoresis was finished, proteins were transferred to a preactivated polyvinylidene fluoride membrane using a Transblot (Biorad) at 20V. For immunodetection, the membrane was blocked with 5% milk and then it was incubated with the following primary antibodies: mouse anti-Nrf-2 (60 KDa) (1/250 dilution; Santa Cruz Biotechnology, Heidelberg, Germany); rabbit anti-HO-1 (32 KDa) (1/1000 dilution; Enzo Life Sciences, New York, NJ, USA) at 4 °C overnight; and mouse anti-tubulin (50 KDa) (1/1000 dilution; Sigma-Aldrich, San Luis, CA, USA) for 1 h at room temperature. Afterwards the membrane was incubated with secondary antibodies linked to horseradish peroxidase (HRP) (anti-mouse (Sigma-Aldrich, San Luis, CA, USA) and anti-rabbit (Sigma-Aldrich, San Luis, CA, USA), respectively, 1/1000 dilution) for 1 h at room temperature. The detection of antigen–antibody binding was assayed by ECL chemiluminescent substrate (Millipore) using an Amersham Imager 600 instrument (Amershan, Buckinghamshire, UK). Protein band intensities were quantified by densitometry with ImageQ image processing software. The relative expression of targeted protein was calculated and normalized by the values of its related tubulin band intensity.

### 4.12. Measurement of β-Galactosidase Activity

Cells were incubated in a 24-well plate (40,000 cells/well) and stimulated with etoposide and the crude fucoidans for 48 h. Fluorescein di-β-galactopyranoside (FDG) was added 1 h previous to harvesting cells. Afterwards, cells were incubated with the pH modulator bafilomycin (Sigma-Aldrich, San Luis, CA, USA) for the last 30 min. Plates were then washed with PBS and cells were collected with trypsin, centrifugated, and subsequently resuspended in PBS. 

FDG is a fluorophore and a substrate for β-galactosidase. The activity of this enzyme is a well-known senescence marker used in several studies [43]. The resulting fluorescence from the reaction of β-galactosidase with FDG was measured by a FACSCalibur flow cytometer (Becton Dickinson) in fluorescence channel 1 and expressed as percentage of positive cells.

### 4.13. Cell-Proliferation Assay

Cells were incubated in a 96-well plate (10,000 cells/well) and stimulated as indicated for 48 h. Cell proliferation was measured employing the BrdU Cell-Proliferation Assay Kit (Cell Signaling Technology), which evaluated the incorporation of 5-bromo-2′-deoxyuridine (BrdU), a thymidine analog, according to the manufacturer’s instructions. 

### 4.14. Measurement of ROS Production

Cells were incubated in a 24-well plate (40,000 cells/well) and stimulated with etoposide and the crude fucoidans for 24 h, and 2′,7′-diclorodihidrofluorescein (DCFDA), a fluorescent redox probe, was added for the last 30 min. Afterwards, plates were washed with phosphate-buffered saline (PBS), and cells were collected with trypsin, centrifugated, and subsequently resuspended in PBS. DCFDA was used to evaluate the production of ROS among the different conditions by flow cytometry, since it passes through the cell membrane and reacts with ROS to generate molecules that have high fluorescence. Fluorescence intensity was measured in fluorescence channel 1 of a FACSCalibur flow cytometer (Becton Dickinson, Mountain View, CA, USA) and expressed as median fluorescence intensity.

### 4.15. Statistical Analysis

Data are presented as the mean of “n” experiments (exact independent sample number “n” for each experiment is shown in the figure) ± standard error of the mean (SEM) or as representative results, as indicated. Results were analyzed using the GraphPad PRISM version 8 statistical software (La Jolla, CA, USA). Differences between experimental conditions were determined by Friedman (multiple comparison) or Wilcoxon (paired comparison) non-parametric tests. *p* ≤ 0.05 was considered statistically significant. 

## 5. Conclusions

In this study we have demonstrated that crude fucoidans from brown algae *Undaria pinnatifida* (Up-MAE) and *Sargassum muticum* (Sm-US, Sm-PHW) obtained by different methods are capable of differently alleviating pathological processes characteristic of OA pathophysiology. After ensuring the crude fucoidans were not toxic for chondrocytes, we proved their anti-inflammatory effects. Findings suggest that these protective actions might be, at least partially, due to its capacity to activate the Nrf-2/HO-1 antioxidant pathway and reduce ROS production. However, we found no evidence that it attenuates OA-associated cellular senescence. Despite some experiments that have been carried out in primary chondrocytes, most have been carried out in a chondrocyte cell line. Thus, further research with more complex models is needed to confirm what we have observed here, determine the best effective dose, and prove the clinical efficacy of these crude fucoidans. 

## Figures and Tables

**Table 1:** Fundamental composition of crude fucoidans tested in this work.

Sample/Composition (%, g/100 g)	Sm-PHW	Sm-US	Up-MAE	Up-C
Glucose	7.06 *	2.88 *	2.75 *	-
Gal+Xyl+Man	28.13 *	10.45 *	10.64 *	24.78 *
Rhamnose	-	0.84 *	0.96 *	-
Fucose	29.69 *	8.79 *	11.40 *	27.10 *
Formic acid	1.28 *	-	1.84 *	-
Acetyl group	1.57 *	-	2.57 *	-
Galacturonic acid	7.05 *	-	-	-
Fuc:Gal+Xyl+Man:Glu	1:0.94:0.24	1:1.19:0.33	1:0.93:0.24	1:0.91:0
Sulphate content	3.28 ± 0.01	37.57 ± 0.01 mg/g	17.01 ± 0.91 mg/g	384.44 ± 1.93 mg/g
Phloroglucinol	3.22 ± 0.01	2.41 ± 0.02	3.99 ± 0.12 mg/g	4.26 ± 0.04
TEAC value	1.29 ± 0.01	4.11 ± 0.01	7.37 ± 0.82 mg/g	5.36 ± 2.09
Reference	[39]	[40]	[38]	Current work

* Standard deviations were lower than 10% for parameters labeled with an asterisk as superscript. Gal: galactose, Xyl: xylose, Man: mannose, TEAC: Trolox equivalent antioxidant capacity.

**Table 2:** Sequences of the primers used to analyze gene expression.

Gene	Reference Sequence	Forward Primer	Reverse Primer
CBS	NM_000071.3	5′-AGGAGAAGTGTCCTGGATGC-3′	5′-TAGGTTGTCTGCTCCGTCTG-3′
CTH	NM_001902.6	5′-GCATTTCAAAAACGGAATGG-3′	5′-CTCATGCTGTGGATGAGAGG-3′
HMOX1	NM_002133.3	5′-TCCGATGGGTCCTTACACTC-3′	5′-TAAGGAAGCAGCAAGAGA-3′
SOD-2	NM_000636.4	5′-CTGGACAAACCTCAGCCCTA-3′	5′-TGATGGCTTCCAGCAACTC-3′
IL-6	NM_000600.5	5′-GATGAGTACAAAAGTCCTGATCCA-3′	5′-CTGCAGCCACTGGTTCTGT-3′
IL-8	NM 000584.3	5′-GAGCACTCCATAAGGCACAAA-3′	5′-ATGGTTCCTTCCGGTGGT-3′
HPRT	NM_000194.3	5′-TGATAGATCCATTCCTATGACTGTAGA-3′	5′-CAAGACATTCTTTCCAGTTAAAGTTG-3′

## Data Availability

The data used to support the findings of this study are contained within the article.

## Abbreviations

^1^H NMR	Proton nuclear magnetic resonance
BrdU	5-bromo-2′-deoxyuridine
CBS	Cystathionine β-synthase
cDNA	Complementary desoxyribonucleic acid
CTH	Cystathionine γ-lyase
DCFDA	2′,7′-Dichlorodihydrofluorescein diacetate
DMEM	Dulbecco’s modified eagle medium
ECM	Extracellular matrix
ELISA	Enzyme-linked immunosorbent assay
FBS	Fetal bovine serum
FDG	Fluorescein di-β-galactopyranoside
FTIR	Fourier transform infrared spectroscopy
HO-1	Heme oxygenase-1
HPRT	Hypoxanthine phosphoribosyl transferase
HPSEC	High-performance size exclusion chromatography
HRP	Horseradish peroxidase
IL-1β	Interleukin 1β
IL-6	Interleukin 6
IL-8	Interleukin 8
MAE	Microwave-assisted extraction
MMPs	Metalloproteinases
mRNA	Messenger ribonucleic acid
MTT	3-(4,5-dimethylthiazol-2-yl)-2,5-diphenyltetrazolium bromide
NSAIDs	Nonsteroidal anti-inflammatory drugs
Nrf-2	Nuclear factor erythroid-2 related factor
OA	Osteoarthritis
P/S	Penicillin/streptomycin
PHW	Pressurized hot-water extraction
PBS	Phosphate-buffered saline
PMSF	Phenylmethylsulfonyl fluoride
qPCR	Quantitative polymerase chain reaction
RNA	Ribonucleic acid
ROS	Reactive oxygen species
SASP	Senescence-associated secretory phenotype
SDS	Sodium dodecylsulfate
sem	Standard error of median
SEM	Scanning electron microscope analysis
Sm	*Sargassum muticum*
TNF-α	Tumor necrosis factor α
Up	*Undaria pinnatifida*
US	Ultrasound-assisted extraction
